# Conductive Elastomers for Stretchable Electronics, Sensors and Energy Harvesters

**DOI:** 10.3390/polym8040123

**Published:** 2016-04-05

**Authors:** Jin-Seo Noh

**Affiliations:** Department of Nano-Physics, Gachon University, 1342 Seongnamdaero, Sujeong-gu, Seongnam-si, Gyeonggi-do 461-701, Korea; jinseonoh@gachon.ac.kr; Tel.: +81-31-750-5611

**Keywords:** conductive elastomers, stretchability, electrical conductivity, PDMS, PU, PEDOT:PSS, PANI, PPY, CNTs, graphene

## Abstract

There have been a wide variety of efforts to develop conductive elastomers that satisfy both mechanical stretchability and electrical conductivity, as a response to growing demands on stretchable and wearable devices. This article reviews the important progress in conductive elastomers made in three application fields of stretchable technology: stretchable electronics, stretchable sensors, and stretchable energy harvesters. Diverse combinations of insulating elastomers and non-stretchable conductive materials have been studied to realize optimal conductive elastomers. It is noted that similar material combinations and similar structures have often been employed in different fields of application. In terms of stretchability, cyclic operation, and overall performance, fields such as stretchable conductors and stretchable strain/pressure sensors have achieved great advancement, whereas other fields like stretchable memories and stretchable thermoelectric energy harvesting are in their infancy. It is worth mentioning that there are still obstacles to overcome for the further progress of stretchable technology in the respective fields, which include the simplification of material combination and device structure, securement of reproducibility and reliability, and the establishment of easy fabrication techniques. Through this review article, both the progress and obstacles associated with the respective stretchable technologies will be understood more clearly.

## 1. Introduction

The demand for stretchable devices has been ever growing since new technology fields like stretchable electronics, intelligent robotics, wearable devices, and body-conformable devices have emerged [1,2,3,4]. For instance, keen sensory skins are required to implement advanced robots that can interact well with humans and properly react to the environment without external control [5,6,7]. Keeping pace with this growing need for new technology, a wave of searching for new materials that can afford high electrical conductivity and also good mechanical elasticity has surged [8,9,10]. Although a variety of conducting polymers, such as polyacetylene (PA), polyaniline (PANI), polypyrrole (PPY), and poly(3,4-ethylene dioxythiophene) (PEDOT) have been developed for diverse applications [11,12,13,14,15], their wide-spread use is limited by their poor mechanical properties. For example, a poly(3,4-ethylene dioxythiophene):poly(styrene sulfonic acid) (PEDOT:PSS) film, which is widely employed in plastic electronics and organics-based optoelectronic devices, shows high electrical conductivity up to 1000 S/cm, but its breaking strain is below 10% [16]. This level of tolerance to strain is not acceptable for the above-mentioned applications.

On the contrary, elastomers such as natural rubber (NR), styrene butadiene rubber (SBR), ethylene-propylene-diene monomer (EPDM), polyurethane (PU), and poly(dimethylsiloxane) (PDMS) are characterized by high, reversible deformation (>200%) and poor conductivity [17,18,19,20]. Traditionally, they have been used mainly for industrial, structural, and household products, and various fillers have been incorporated into them to reinforce mechanical properties like tensile strength and Young’s modulus [21,22,23,24,25]. Interestingly, some conductive fillers typified by a family of carbon matters, such as carbon blacks, graphites, and carbon nanotubes (CNTs) have been introduced to transform the resin from an insulator to a conductor [26,27,28]. However, this approach requires a significant amount of filler to be added, causing a drastic loss of the material’s elasticity, even though the degradation of elasticity can be minimized for CNT-elastomer composites, where conductive CNT percolation networks can be formed by the addition of just a small amount of CNTs.

For the sake of the simultaneous satisfaction of high electrical conductivity and good elasticity, some groups have attempted to modify the molecular structures of elastomers. Doping SBR with iodine (I_2_) and antimony pentachloride (SbCl_5_) [29,30], and synthesizing graft copolymers composed of PU and PANI are typical examples [31,32]. However, these approaches require elaborate adjustment of experimental conditions and are yet to confirm their long-term reliability. A more practical and easier way of tackling the goal is to make a blend consisting of a conducting polymer and an elastomer [33,34,35]. In this scheme, the conducting polymer plays a role in increasing the conductivity of the blend, while the elastomer renders the material stretchable. When 11.5% of PANI by volume fraction was added to PU, the material’s conductivity increased by six orders of magnitude while maintaining high stretchability of 200% [34]. For a PEDOT–PU blend, a high conductivity of 100 S/cm was demonstrated, even under an elongation of >100% [35]. In general, the electrical conductivity of a blend increases at the cost of mechanical properties as the fraction of conductive part increases. Thus, elaborate material design is necessary to fulfill detailed specifications required for specific applications.

In this review, diverse efforts to realize conductive elastomers and recent progress are discussed. Owing to the massiveness of prior works, some representative results in the fields of electronics, sensors, and energy harvesting will be introduced. It is noteworthy that similar strategies and similar material combinations have sometimes been adopted in different fields of application. Through this review, not only achievements made till now, but tasks to be solved will also be considered.

## 2. Stretchable Electronics

The scope of stretchable electronics is very wide, including stretchable logic gates, memories, and stretchable display units. Although the respective devices have different structures, they are generally organized with basic elements, such as interconnects, transistors, light-emitting diodes (LEDs), and dielectrics. One of the key strategies for implementing stretchable electronics is a combination of rigid, active components and stretchable interconnects, which accommodate external strains [36,37,38]. However, to realize genuine stretchable electronics, all the components need to be stretchable. From the aspect of materials, this requires conductors, semiconductors, and dielectrics, all of which are stretchable. In this section, stretchable conductors and a couple of representative stretchable devices are briefly reviewed.

### 2.1. Stretchable Conductors

Two types of metal structures (*i.e.*, wavy structures and serpentine structures) have been widely explored as stretchable conductors. Wavy metal structures are formed from metal films deposited on strained or pre-patterned elastomeric substrate [38,39,40,41], and their reversible stretchability is relatively low (<20%). On the other hand, serpentine metal structures are 2D or 3D arrays of a repeating horseshoe-shaped unit [42,43,44]. Hsu *et al.* demonstrated the ultimate elongation of 250% and the reliability of 40,000 cycles at a 30% elongation, using polyimide-enhanced Cu serpentine structures [44]. Although these methods proved their effectiveness, they usually require a multi-step process including a costly lithography step.

**Figure 1:**
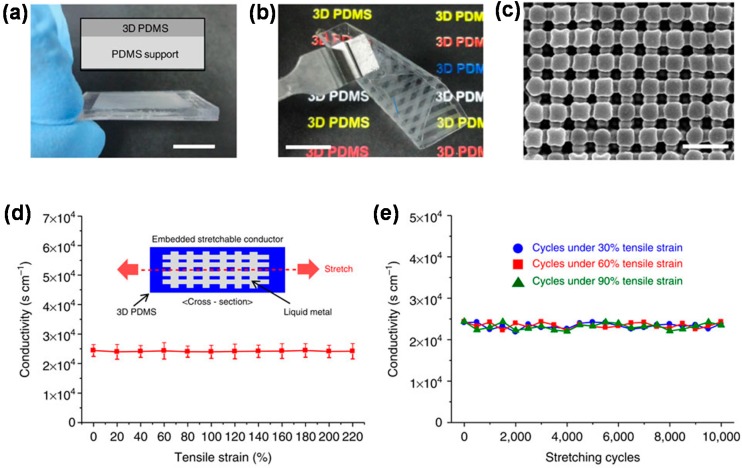
Optical images of (**a**) a 3D poly(dimethylsiloxane) (PDMS) film on PDMS support and (**b**) a folded 3D PDMS film. Scale bar, 1 cm; (**c**) Top-view SEM image of net-shaped 3D PDMS film. Scale bar, 1 μm; (**d**) Conductivity of 3D PDMS–eutectic gallium–indium (PDMS–EGaIn) stretchable conductor under strains of up to 220%; (**e**) Conductivity variation depending on the number of stretching–releasing cycles under different strains. Reproduced with permission from [55]. Copyright 2012 Macmillan Publishers Limited.

Another approach is to employ elastomeric composites. The elastomeric composites may be elastomer-conducting polymer blends, elastomeric composites incorporating carbon nanostructures or metal nanostructures, and polymer blends incorporating nanostructures of conductive materials [45,46,47,48,49,50,51,52]. Almost two decades ago, Fu *et al.* prepared elastomeric PU—PPY composite foams by *in situ* polymerization of pyrrole in pre-formed PU foams, and a conductivity of 10^−5^ S/cm and an elongation at break of 160% were obtained from a 6 wt % PPY-containing composite [53]. The low conductivity of the composite, which was one of the main issues with techniques using PU foams, has been improved by introducing conductive nanostructures. For example, Ge *et al.* fabricated PU sponge–Ag nanowire–PDMS stretchable conductors by a simple solution-dipping method, and confirmed high conductivity of 19.2 S/cm and a resistance change *(*∆*R*/*R*_0_) of 160% at a 100% strain [54]. Instead of PU foams, Park *et al.* constructed 3D PDMS structures using proximity-field nanopatterning, and produced stretchable, transparent conductors by infiltrating liquid metal, eutectic gallium–indium (EGaIn), into the 3D PDMS (Figure 1a–c) [55]. The 3D PDMS-EGaIn stretchable conductors showed extremely high conductivity (24,100 S/cm), even under strains of >200%, and good stretching cycle performance (Figure 1d,e).

Hansen *et al.* synthesized PU-PEDOT blends from liquid mixtures of EDOT and varying amounts of PU dissolved in tetrahydrofuran (THF) without engaging porous elastomers [35]. They reported a good conductivity of 10–50 S/cm at a 200% strain for the blends. As a similar strategy, Noh produced PDMS-PEDOT:PSS blends by introducing a miscibility-enhancing copolymer, poly(dimethylsiloxane-*b*-ethylene oxide) (PDMS-*b*-PEO), and demonstrated a conductivity up to 2 S/cm and a fracture strain of 75% [56]. Single-walled CNTs (SWCNTs) and multi-walled CNTs (MWCNTs) have been very widely-employed to transform insulating elastomers to conductors. Kim *et al.* prepared SWCNT–PDMS composites by backfilling SWCNT aerogels, and observed conductivities of 70–108 S/m and a small resistance change of 14% at a tensile strain of 100% [57]. Shin *et al.* utilized a similar approach, in which aligned MWCNTs were first prepared by catalyst-assisted chemical vapor deposition (CVD) and subsequently infiltrated by PU solution [58]. The resulting MWCNT–PU composites showed a conductivity of 50–100 S/m and reversible resistance change for strains up to 40%. Graphene, another material in the carbon family, has been increasingly applied for stretchable conductors through a smart combination with appropriate elastomers [48,51,59]. According to Lee *et* *al.*, for instance, a composite of functionalized graphene sheets and PU could reach an elongation at break of 374% while retaining a conductivity of 1.2 × 10^−5^ S/cm [51].

Silver (Ag) nanostructures have also been intensively investigated as a conductivity-boosting component for stretchable conductors. Xu and Zhu reported that their Ag nanowires (AgNWs)-embedded PDMS composite achieved a high conductivity of 5285 S/cm in a tensile strain range of 0%–50% [60]. Lee *et al.* formed networks of very long AgNWs on Ecoflex using a vacuum filtration and transfer method, and they exhibited a sheet resistance of 9–70 Ω/sq, high transparency of 90%–96%, and good stretchability of >460% [61]. Araki *et al.* prepared Ag flakes-PU composites by emulsion mixing, and achieved a low resistivity of 2.8 × 10^−4^ Ω·cm and high stretchability up to 600% [62]. In another interesting approach, PU-gold nanoparticle (AuNP) composites were made by either layer-by-layer assembly or vacuum-assisted flocculation, and they showed a maximum conductivity of 11,000 S/cm and stretchability of 486% [63]. Moreover, AuNPs in these composites could be reorganized under stress, allowing for electronic control over mechanical properties.

### 2.2. Stretchable Field-Effect Transistors and Memories

The common methods of fabricating stretchable field-effect transistors (FETs) include the combined use of rigid gate stack and stretchable conductors, adoption of wavy structures of inorganic materials, and realization of organic or composite FETs composed of fully stretchable components. Shin *et al.* fabricated FET arrays of suspended SnO_2_ NWs with wavy interconnects, and demonstrated high stretchability up to 40% and current on/off ratios of 10^6^ [64]. Sekitani *et al.* fabricated large-area stretchable active matrix consisting of 19 × 37 organic transistors, incorporating a SWCNT-based elastomeric conductor [65]. The device could be stretched up to 70% both uniaxially and biaxially without mechanical fracture. Furthermore, Kim *et al.* produced stretchable complementary metal-oxide-semiconductor (CMOS) inverters and three-stage ring oscillators on PDMS [38]. Those devices adopting wavy structures of single-crystalline silicon (Si) nanoribbons showed high gains of 100 and stable oscillation frequency of ~3 MHz, even under a 5% strain.

**Figure 2:**
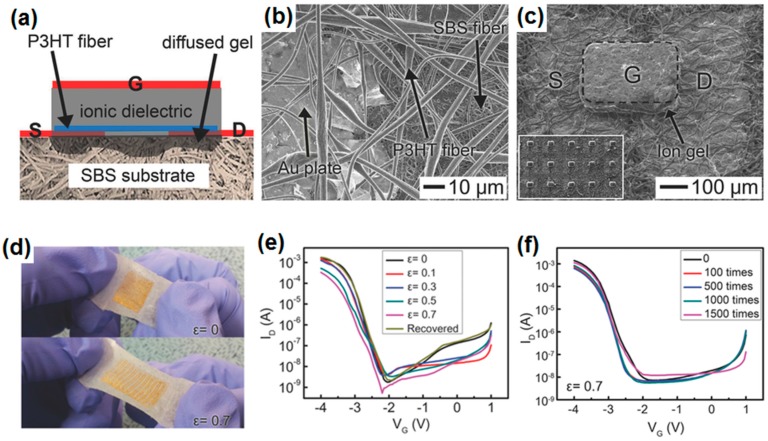
(**a**) Schematic picture; (**b**) focused SEM image; and (**c)** low-magnification SEM image of a high stretchable transistor consisting entirely of stretchable components; (**d**) Photo images of the stretchable transistor array at two strain states (ε = 0 and 0.7); *I*_D_–*V*_G_ curves depending on (**e**) the applied strain and (**f**) the number of stretching–release cycles at ε = 0.7. In (**a**) and (**c**), S, D, and G represent source, drain, and gate, respectively. Reproduced with permission from [66]. Copyright 2014 John Wiley and Sons. P3HT: poly(3-hexylthiophene); SBS: poly(styrene-*b*-butadiene-*b*-styrene).

Recently, Jeong’s group developed stretchable transistors made entirely of stretchable components [66]. They used a poly(styrene-*b*-butadiene-*b*-styrene) (SBS) fiber mat as an elastomeric substrate. Au nanosheets, polyelectrolyte gel, and poly(3-hexylthiophene) (P3HT) nanofibers were employed for electrodes, gate dielectric, and active channel, respectively. The detailed device structures are shown in Figure 2a–c. Au nanosheet electrodes were formed through a transfer process using PDMS pillars, and P3HT fibers were electrospun on the substrate across source and drain electrodes. The transistors were reversibly stretched up to a 70% strain (ε = 0.7), as demonstrated in Figure 2d. Not only mechanically, but also electrically, the transistors exhibited reproducible performance up to 1500 cycles of stretching–recovery at ε = 0.7 (Figure 2e,f). The transistors showed a high hole mobility of 18 cm^2^/V·s and an on/off ratio of 10^5^ even under ε = 0.7.

**Figure 3:**
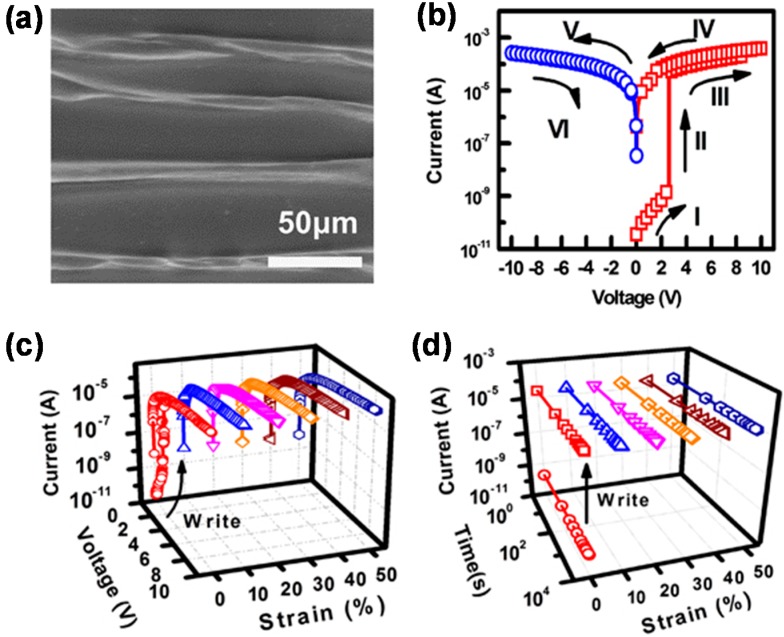
(**a**) Top-view SEM image of the wrinkled organic memory; (**b**) Current–Voltage characteristics showing a memory behavior; Strain-dependent (**c**) memory switching and (**d**) retention time. Reproduced with permission from [69]. Copyright 2014 Nature Publishing Group.

Compared to stretchable transistors, stretchable memories have been less investigated. Flexible memories have been developed as a key component of flexible electronics. For example, Ouyang *et al.* made organic resistive memories from a polystyrene film containing AuNPs and 8-hydroxyquinoline that were sandwiched between two metal electrodes [67]. Ji *et al.* fabricated a twistable memory cell array adopting one diode-one resistor (1D-1R) structure and various organic materials for diode and resistor components [68]. Although their memory cell array stably showed high on/off ratios of >10^3^ up to a twisting angle of 30°, it was broken at a strain of 2.03%. Lai *et al.* developed a stretchable organic memory with a buckled structure, where a combination of wrinkled graphene bottom electrode and polymer compound was used [69]. After a blend of poly(methylmethacrylate) (PMMA) and poly(3-butylthiophene) (P3BT), which played as the active information storage, was spin-coated on a CVD-grown graphene sheet, the film stack was transferred on the pre-strained PDMS substrate. This resulted in a wrinkled organic memory structure, as shown in Figure 3a. This memory switched from a low-current state (“0”) to a high-current state (“1”) at a threshold voltage of 2.6 V, and the state was stably maintained even after the removal of the applied voltage, which is a typical feature of non-volatile memory (Figure 3b). As presented in Figure 3c,d, the memory behavior was not deteriorated by a strain up to 50% and the data retention reached 10^4^ s.

### 2.3. Stretchable Light-Emitting Diodes

**Figure 4:**
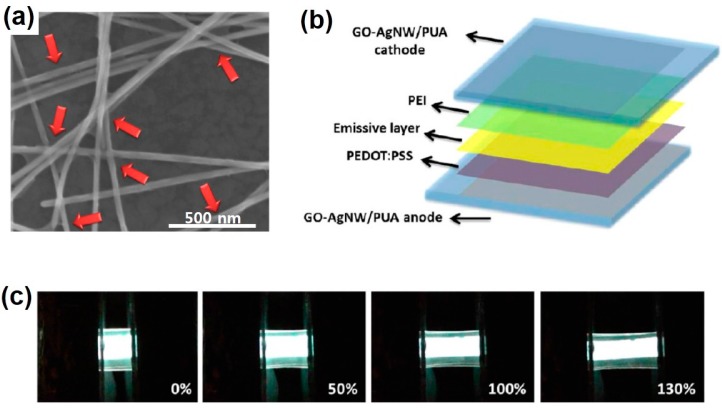
(**a**) SEM image of graphene oxide (GO)-soldered silver nanowire (AgNW) junctions. Red arrows indicate GO parts wrapping around AgNW junctions; (**b**) Schematic drawing of a stretchable PLED structure; (**c**) Optical photographs of a polymer light-emitting device (PLED) operating at 14 V under different strains. Reproduced with permission from [74]. Copyright 2014 American Chemical Society. PEI: polyethylenimine; PEDOT: poly(3,4-ethylene dioxythiophene); PSS: poly(styrene sulfonic acid); PUA: poly(urethane acrylate).

The need for stretchable light-emitting systems is ever increasing in fields such as wearable displays, rollable lamps, and biocompatible light sources. A common way of implementing stretchable displays is to combine elastic interconnects with rigid inorganic or organic LEDs [70,71]. Another approach for stretchable displays is to adopt intrinsically-stretchable organic LEDs (OLEDs), where all the constituents are stretchable. Filiatrault *et al.* fabricated light-emitting electrochemical cells (LEECs) using stretchable ruthenium (Ru)/PDMS emissive layers and stretchable Au/PDMS anodes [72]. Even though they demonstrated large-area emission, the strain tolerance and the external quantum efficiency of the devices were relatively low. Pei’s group developed transparent stretchable electrodes made of AgNW-poly(urethane acrylate) (PUA) composite, and fabricated elastomeric polymer light-emitting device (EPLED) by combining them with electroluminescent polymer layer [73]. The EPLED could emit light even at a high strain of 120%. The same group improved the performance of transparent stretchable electrodes by introducing graphene oxide (GO) to AgNW percolation networks [74], as shown in Figure 4a. The GO soldering turned out to reduce AgNW junction resistance and suppress the inter-NW slip. The group fabricated PLED composed of GO–AgNW–PUA composite electrodes, a polymeric emissive layer, and a polyethylenimine (PEI) electron transporting layer (Figure 4b). The PLED could be stretched up to 130% (Figure 4c) and endure over 100 stretching cycles between 0%–40%.

### 2.4. Brief Summary

Representative achievements made in the stretchable electronics field are summarized in Table 1.

Of the three applications in this field, stretchable conductors have made the greatest advance. They have achieved extremely large stretchability, up to 600%, while keeping a high electrical conductivity of more than 10^3^ S/cm, which is the level required for conventional electrodes and interconnects. However, easier methods to fabricate such high-performance stretchable conductors need to be developed. Stretchable FETs have demonstrated good on/off ratios larger than 10^6^, but their stretchability still needs to be further improved for the fully stretchable applications. Stretchable memories are in their infancy. Stretchable LEDs have made a big progress in terms of stretchability, while their external quantum efficiency (EQE) is still far lower than their inorganic counterparts.

## 3. Stretchable Sensors

Although there are a wide variety of sensors that have been devised to sense a large number of stimuli or materials, they can be divided into two categories: physical sensors and chemical sensors. Physical sensors sense physical stimuli such as pressure, strain, and temperature, whereas chemical sensors detect chemical species in gas phase or liquid phase. Despite this dissimilarity in sense targets, the two groups of sensors take advantage of similar sensing principles, which are based on a change in the physical properties of a sensing material or device, such as electrical resistance, capacitance, and optical reflectance [75,76,77,78]. In the field of sensors, the demand for stretchable sensors has been growing more and more for special applications such as body-conformable or implantable health monitors, wearable sensory textiles, and electronic skins for intelligent robots.

### 3.1. Stretchable Strain Sensors

Strain sensors are devices to precisely detect various mechanical deformations like elongation, compression, and bending. In the traditional field of structural health monitoring, relatively low stretchability of below 1% would be enough for application. However, for stretchable strain sensors that are the focus here, high stretchability is required in addition to conventional sensor requirements such as high sensitivity, fast response, and good stability. Conventional metal-based strain sensors show the maximum strain of only about 5% [79] and a gauge factor of ~2 [80]. Graphene sheets coated on polymeric substrates have attracted great interest, especially in the aspect of sensitivity [81,82,83]. Even though they have achieved a colossal gauge factor of ~10^6^, their stretchability was not good (<10%) [82,83]. In contrast, CNT films on elastomeric substrates have exhibited large stretchability. For example, Fan *et al.* fabricated strain sensors from CNT networks coated on PU multifilaments, and showed a large, reversible stretchability of 400% [84]. Yamada *et al.* fabricated vertically aligned SWCNT films on PDMS substrate, and demonstrated high stretchability of 280% and low creep of 3% at a 100% strain [85]. Despite the high stretchability, the gauge factor of the CNT-elastomer composites was relatively low (<5). As another approach for realizing stretchable strain sensors, some research groups have used cracked metal film structures on an elastomeric substrate. Noh formed microcracks and buckle structures in titanium (Ti) films sputtered on PDMS by mechanical stretching [86], and Lacour *et al.* induced tri-branched microcracks in Au films electron-beam-evaporated on PDMS [87]. Although these strain sensors were easily fabricated and presented strain-dependent resistance change, the repeatability of resistance signals needs to be further guaranteed.

**Figure 5:**
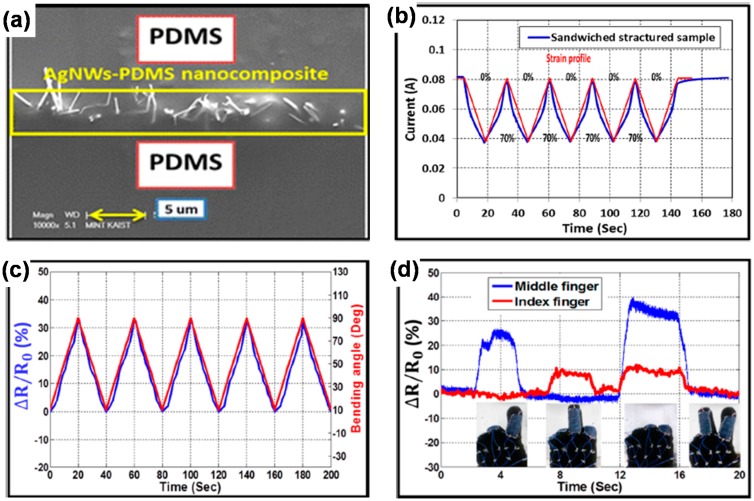
(**a**) Cross-sectional SEM image of PDMS–AgNWs–PDMS sandwich-structured strain sensor. Repeated responses of the strain sensor (**b**) to stretching–releasing cycles at a strain of 70% and (**c**) to bending cycles in the bending angles of 10°–90°; (**d**) Demonstration of finger motion detection using the strain sensor. Reproduced with permission from [89]. Copyright 2014 American Chemical Society.

AgNW-elastomer composites have also been actively applied for stretchable strain sensors [60,88,89]. Xu and Zhu produced a stretchable capacitive strain sensor by embedding AgNW films on both surfaces of a PDMS sheet [60]. The capacitance of the sensor changed linearly and reproducibly with the applied strain in the range of 0%–50%. However, its gauge factor was merely ~1. Amjadi *et al.* implemented a stretchable resistive strain sensor, taking a similar approach [89]. Their PDMS-AgNWs-PDMS sandwich-structured strain sensor (Figure 5a) showed reproducible resistance changes under a range of linear strain of 0%–70% and even under a bending strain (Figure 5b,c). This strain sensor showed almost no hysteresis up to a strain of 40%, and hysteresis behaviors appeared at a 60% strain with full resistance recovery at zero strain. Human motion detection was also demonstrated using the strain sensor (Figure 5d).

### 3.2. Stretchable Pressure Sensors

Unlike strain sensors, which mostly detect lateral deformations, pressure sensors detect the magnitude of force vertically acting on a unit area of plane. The main method for the implementation of stretchable pressure sensors is to combine a conductive matter with an elastomer in a composite or in a specifically-devised structure. In the approaches using composite materials, conductive nanostructures such as metal nanoparticles/nanowires, carbon blacks, and CNTs are distributed through an insulating elastomer [90,91,92], and a resistive change under a pressure is measured. However, uniform distribution of conductive nanostructures is yet to be confirmed more. As a slightly different approach, Brady *et al.* coated a PU foam with PPY by soaking the PU foam in a solution containing pyrrole monomer and naphthalene di-sulphonic acid followed by *in situ* polymerization [93]. They demonstrated real-time monitoring of ribcage movement while breathing, using this PPY-coated PU foam.

**Figure 6:**
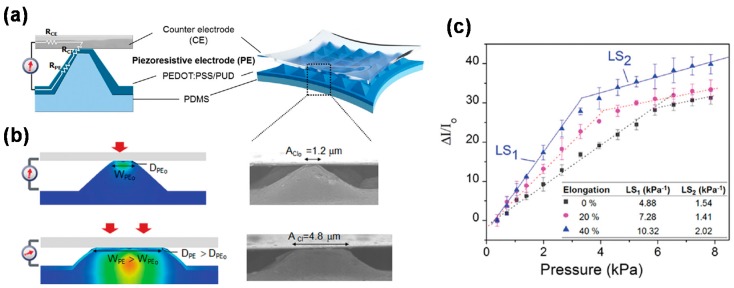
(**a**) Schematic picture of a micro-pyramid PDMS array. Individual PDMS pyramids are coated with a PEDOT:PSS-PUD (Polyurethane dispersion) blend, which serves as a piezoresistive electrode; (**b**) Finite element analysis data showing stress distributions and SEM images at different magnitudes of pressures; (**c**) Relative current changes depending on the applied pressure while the sensor is stretched to a certain elongation. Here, LS_1_ and LS_2_ represent the linear sensitivities in the respective regions. Reproduced with permission from [96]. Copyright 2014 John Wiley and Sons.

More recently, several research groups have developed special device structures to achieve highly-sensitive pressure sensors. Joo *et al.* fabricated a capacitive pressure sensor consisting of an AgNW-embedded PDMS electrode and a PMMA dielectric layer [94]. Despite its high sensitivity (>3.8 kPa^−1^), however, it was not stretchable. Park *et al.* fabricated a stretchable pressure sensor that consisted of Au-coated PDMS micropillars (top layer) and PANI nanofibers (bottom layer) [95]. This sensor operated by measuring a change of contact resistance between the two layers under an applied pressure. They obtained a high sensitivity of 2.0 kPa^−1^, a low detection limit of 15 Pa, a fast response time of 50 ms, and a biaxial stretchability of 15% from this sensor. To improve the sensitivity more, Choong *et al.* developed a micro-pyramid PDMS array [96]. As shown in Figure 6a, the PDMS micro-pyramids were coated with a PEDOT:PSS-PUD (PU dispersion) blend, which functioned as a piezoresistive electrode. Upon the application of pressure, each pyramid tends to spread laterally, and this increases the contact interface area (*A*_CI_), contact perimeter (*W*_PE_), and the thickness of current path (*D*_PE_), thereby increasing the current conduction (Figure 6b). This sensor worked fine even at a high elongation of 40%, and showed a very high sensitivity of 10.32 kPa^−1^ at that elongation (Figure 6c).

### 3.3. Stretchable Temperature Sensors

**Figure 7:**
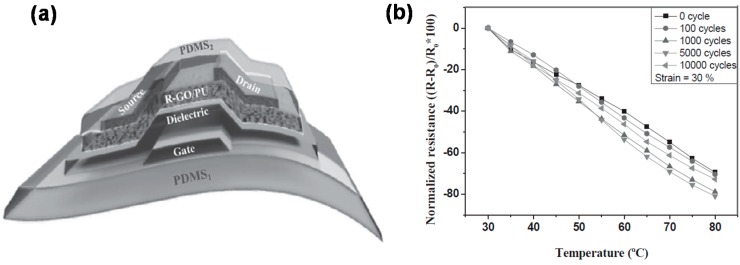
(**a**) Schematic drawing of all elastomeric gated temperature sensor; (**b**) Response of the temperature sensor after cyclic stretching of 0 to 10,000 cycles at a strain of 30%. Reproduced with permission from [100]. Copyright 2016 John Wiley and Sons. R-GO: Reduced Graphene Oxide.

Stretchable temperature sensors have attracted growing attention, especially for body-attachable applications. The traditional resistance temperature detector (RTD) takes advantage of the principle that a conductor’s resistance changes with temperature. However, good metals like Au and platinum (Pt) that are commonly used for temperature sensors do not have stretchability by themselves. To overcome this shortcoming, Chen *et al.* used a serpentine structure of Au and combined it with porous PU [97]. Their temperature sensor showed good linearity of resistance change, good stretchability, and good air-permeability. Likewise, Yan *et al.* used a coiled structure of graphene that was embedded inside PDMS [98]. This graphene thermistor presented nonlinear resistance variation with temperature in the range of 30–100 °C and tolerance to strain up to 50%. Hong *et al.* employed a thin film transistor (TFT) array with a stretchable PANI nanofiber temperature-sensing component [99]. The SWCNT TFTs and PANI nanofiber sensors were first prepared on poly(ethylene terephthalate) (PET) films, and then they were implanted onto Ecoflex. This temperature sensor exhibited a high resistance sensitivity of 1.0% °C^−1^, a response time of 1.8 s, and mechanical stability to biaxial strain up to 30%. Furthermore, Trung *et al.* fabricated an all elastomeric gated temperature sensor [100]. For that, a PEDOT:PSS-PUD composite was used as source, drain, and gate material, PU as a gate dielectric, and reduced graphene oxide (R-GO)-PU composite as a temperature-sensing channel layer (Figure 7a). This temperature sensor could stretch up to a strain of 70% and exhibited a high sensitivity of 1.34% °C^−1^ and stable response to temperature, even after 10,000 cycles of stretching at a 30% strain (Figure 7b).

### 3.4. Brief Summary

Representative achievements made in the field of stretchable sensors are summarized in Table 2.

All three applications have made remarkable achievements. In particular, stretchable strain sensors have shown great performance in both mechanical stretchability and gauge factor, and demonstrated their functionality in the area of motion detection. Simple combinations of conductive nanostructures and elastomers have been proven to work effectively for this application. Stretchable pressure sensors seem to need further improvement in stretchability, although their sensitivity is good enough. Some stretchable temperature sensors have met both requirements of stretchability and temperature sensitivity. However, their structures and material combinations are in general complex, and further efforts to develop simpler systems would be necessary.

## 4. Stretchable Energy Harvesters

Since the global warming issue caused by the use of fossil fuel has surged, new energy generation methods have been in great demand. Various methods of harvesting energy from diverse sources were developed to reduce the consumption of fossil fuel, and some of them have already come into our everyday life. Solar (or photovoltaic) cells that convert solar energy to electrical energy are a typical example. Piezoelectric energy harvesting and thermoelectric energy harvesting have also been expanding their application areas. Traditionally, these energy harvesters were made of inorganic materials like Si, lead zirconate titanate (PZT), and bismuth telluride (Bi_2_Te_3_), all of which are brittle. Due to the abruptly increasing use of portable or wearable low-power-operated electronics, the need for stretchable energy harvesters has been growing larger.

### 4.1. Stretchable Solar Cells

Organic photovoltaic (OPV) cells have been of increasing interest in various applications, particularly for wearable electronics, electronic skin, and intelligent robotics [101,102,103,104]. About a decade after Shaheen *et al.* reported a record-high power conversion efficiency (PCE) of 2.5% for OPV cells [105], Mitsubishi Chemical replaced the record with 9.2% [102]. For the conventional bulk heterojunction OPV cells, indium tin oxide (ITO) is used as transparent electrode and it is coated with a hole transport layer (HTL), typically made of PEDOT:PSS. Over the HTL, active layer that is a mixture of donor and acceptor materials is coated by co-deposition or solution casting method. Up to now, the most efficient OPV cells, the PCE of which is above 3.5%, have come from solution-cast P3HT:PCBM ((6,6)-phenyl-C_61_-butyric acid methyl ester) blends. These material combinations and device structure have been maintained as a basic platform for the realization of stretchable solar cells. A straightforward way of implementing a stretchable solar cell is to connect highly efficient, but rigid inorganic cells with stretchable interconnects. Lee *et al.* first fabricated gallium arsenide (GaAs) microcells on Si substrate and transferred them to PDMS substrate [106]. Then, arc-shaped Au interconnects were transferred to bridge the microcells. This microcell array showed a high fill factor of 0.79 and PCE of 13%, but its stretchability was limited to below 30%.

**Figure 8:**
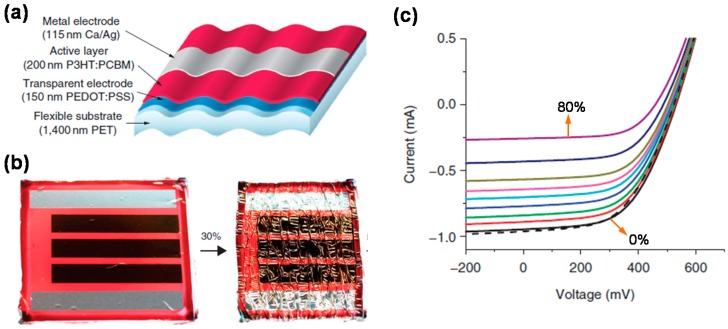
(**a**) Schematic of the ultrathin organic solar cell; (**b**) Stretchable solar cell fabricated simply by attaching the ultrathin cell to a pre-stretched elastomer. It can be compressed and re-stretched; (**c**) Current-voltage curves of the solar cell depending on the compression in the range of 0%–80%. Between the black (0%) and purple (80%) lines, individual colors represent the compression increasing with a step of 10%. The black dashed line represents the device after being restored to its initial state. Reproduced with permission from [108]. Copyright 2012 Nature Publishing Group.

Lipomi *et al.* fabricated stretchable organic solar cells based on organic materials described above [107]. They spin-coated PEDOT:PSS layer and P3HT:PCBM layer and deposited liquid metal sequentially on pre-strained PDMS substrate. Then, buckles were induced to the layer stack upon relaxing the PDMS substrate. This buckle-structured solar cell showed endurance to strain up to 22.2%. However, its PCE (1.2%) and fill factor (0.38) were not good. Using the same material combination for the active layer, Kaltenbrunner *et al.* fabricated an ultrathin OPV cell [108]. As depicted in Figure 8a, the total thickness of the solar cell is only 1.9 μm. When this OPV cell stacked on a PET film was attached to a pre-stretched elastomer, it could tolerate a large compression of up to 80% (Figure 8b). This solar cell exhibited improved performance with a fill factor of 0.61 and a PCE of 4.2%. Although almost restored to the original value after re-stretching it to its initial state, the short circuit current of the solar cell decreased with increasing compression from 0% to 80% (Figure 8c).

### 4.2. Other Stretchable Energy Harvesters

**Figure 9:**
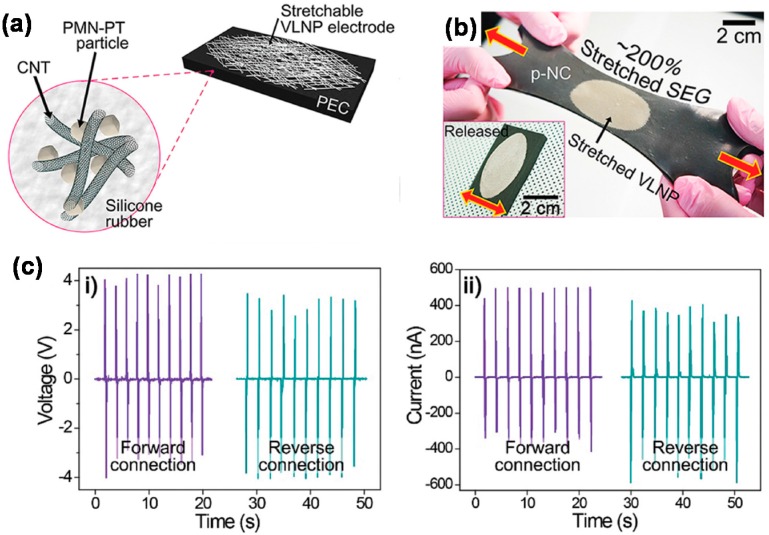
(**a**) Schematic picture of the hyper-stretchable nanocomposite generator (SEG); (**b**) The SEG can be stretched and released without damage; (**c**) Generated (**i**) open-circuit voltage and (**ii**) short-circuit current depending on periodic stretching–releasing cycles at a strain of 200%. Reproduced with permission from [114]. Copyright 2015 John Wiley and Sons. VLNP: very long nanowire percolation; PMN-PT: lead magnesio niobate–lead titanate; PEC: piezoelectric elastic composite.

Piezoelectric energy harvesters convert diverse mechanical stimuli to electricity. Flexible piezoelectric energy harvesters have been reported, which employed various nanostructured materials such as PZT nanowires/ribbons, ZnO nanorods/nanowires, and polyvinylidene fluoride (PVDF) nanofibers on flexible substrates like polyimide and paper [109,110,111,112]. For example, Zhou *et al.* have obtained a high power density of 2.4 μW·cm^−3^ using a PZT NW–PDMS nanocomposite [111]. Although these energy harvesters proved to be highly flexible, they lacked stretchability. Lee *et al.* fabricated a stretchable hybrid nanogenerator (NG) that incorporated PDMS–CNT composite as the bottom electrode, poly(vinylidene fluoride-*co*-trifluoro ethylene) as the energy-harvesting layer, and graphene as the top electrode [113]. Their NG could stretch up to a strain of 30%, but its output power was not large enough, probably due to the insufficient piezoelectricity and pyroelectricity of the harvesting material. Jeong *et al.* demonstrated a hyper-stretchable elastic generator (SEG) by forming stretchable very long nanowire percolation (VLNP) electrodes on a piezoelectric elastic composite (PEC) composed of lead magnesio niobate–lead titanate (PMN–PT) particles and MWCNTs dispersed in a silicone rubber (Figure 9a) [114]. This SEG showed a large, reversible stretchability of ~200% (Figure 9b) and high power output of ~4 V and ~500 nA (Figure 9c).

**Figure 10:**
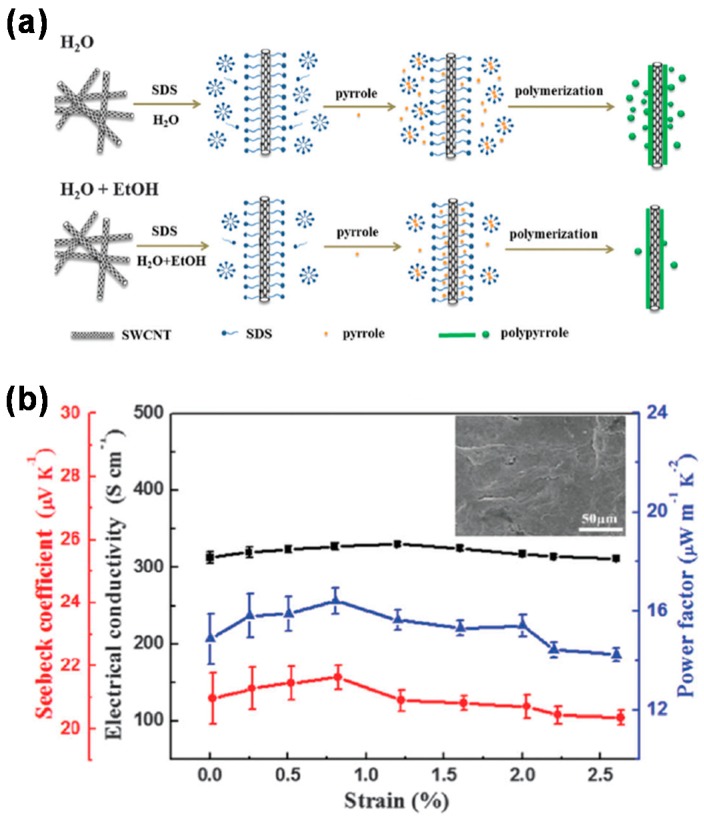
(**a**) Schematic illustration for the preparation procedures of PPY–SWCNT nanocomposites; (**b**) Dependence of thermoelectric performance of the composite on mechanical stretching. The inset is an SEM image of the composite film after a 2.5% stretching. Reproduced with permission from [118]. Copyright 2016 Royal Society of Chemistry.

Thermoelectric energy harvesting is a technique to produce electrical power from waste heat. In recent years, organic thermoelectric generators (TEGs) have gained much attention due to several advantages over their inorganic counterparts, such as low cost, good processibility and flexibility, and low thermal conductivity. The main materials for the organic TEGs were conducting polymers and composites based on them [115,116]. Despite many achievements in flexible organic TEGs, research on stretchable TEGs has been scarce. Kim *et al.* fabricated a wearable TEG consisting of inorganic thermoelectric components formed on glass fabric [117]. Although this TEG embedded in PDMS was thin, flexible, and exhibited a high output power density of 28 mW·g^−1^ at a ∆*T* = 50 K, it was not stretchable. Liang *et al.* took the first step toward a stretchable TEG [118]. They prepared PPY-SWCNT nanocomposites using an *in situ* oxidative polymerization method, as depicted in Figure 10a. As a consequence of the reaction, SWCNTs coated with PPY were obtained, and their films were prepared by vacuum filtration method. This nanocomposite film showed greatly improved thermoelectric performance with a power factor of 19.7 μW·m^−1^·K^−2^, which is the largest value for PPY composites. In addition, it could be stretched by 2.6% (Figure 10b), but its stretchability still needs to be improved.

## 5. Conclusions

Coping with new technology waves, such as wearable/stretchable devices, intelligent robotics, and body-conformable devices, the demand for conductive elastomers has been swirled. As a response to this demand, a variety of research has been intensively undertaken to develop optimal conductive elastomers. Although similar material combination and similar structure have been employed sometimes in different fields of application, detailed strategies altered depending on the target application and a goal. In this article, research efforts put into three fields of stretchable technology, which are stretchable electronics, stretchable sensors, and stretchable energy harvesters, are reviewed. In each field, how conductive elastomers were incorporated into representative devices, how those materials were prepared, and what performance has been achieved with the conductive elastomers are introduced and analyzed. In many cases, insulating elastomers such as PDMS, PU, and silicone rubber were combined either with conducting polymers like PEDOT:PSS, PANI, and PPY, or with conductive nanostructures such as AgNWs, CNTs, and graphene for the implementation of conductive elastomers. Other than the conductive elastomers, stretchable functional materials were also incorporated to endow the key functions of designed devices. P3HT nanofibers for a stretchable FET, a Ru/PDMS emissive layer for a stretchable display, and PMN-PT particles dispersed in PDMS for a stretchable piezoelectric energy harvester are those examples. PDMS has been most widely employed as an elastomeric substrate for most applications. As pronounced achievements, a stretchable conductor has shown stretchability of up to 600% and high conductivity more than 10^3^ S/cm. Also, a stretchable strain sensor has demonstrated stretchability of 400% with a gauge factor of ~5. However, issues such as the reliability of conductive elastomers and the development of easy fabrication techniques are yet to be resolved. Despite the notable achievements in stretchable applications spurred by conductive elastomers, it is also true that many stretchable technologies still need to be further explored.

## Figures and Tables

**Table 1:** Representative achievements made in the field of stretchable electronics.

Application	Material	Mechanical properties	Electrical properties	Reference
Stretchable conductors	PU-PPY composites	ε_b_ = 160%	σ = 10^−5^ S/cm	[53]
	3D PDMS-EGaIn	ε_b_ = 220%	σ = 24,100 S/cm	[55]
	PU-PEDOT blends	ε = 200%	σ = 10–50 S/cm	[35]
	Graphene sheets–PU composites	ε_b_ = 374%	σ = 1.2 × 10^−5^ S/cm	[51]
	Ag flakes–PU composites	ε_b_ = 600%	ρ = 2.8 × 10^−4^ Ω·cm	[62]
Stretchable FETs and memories	SnO_2_ NWs/wavy interconnects	ε_b_ = 40%	On/Off ratio = 10^6^	[64]
	SWCNT–elastomer composites	ε_b_ = 70%	On/Off ratio > 10^3^	[65]
	SBS fiber mat/P3HT nanofibers/polyelectrolyte gel	ε = 70%	On/Off ratio = 10^5^	[66]
	P3HT/PS–PCBM/PEN	ε_b_ = 2.03%	On/Off ratio > 10^3^	[68]
	PMMA–P3BT/PDMS	ε_b_ = 50%	Data retention = 10^4^ s	[69]
Stretchable LEDs	Ru-PDMS/Au–PDMS	ε_b_ = 27%	EQE < 1%	[72]
	AuNW–PUA composites	ε_b_ = 120%	EQE = 4%	[73]
	GO–AgNW–PUA composites/PEDOT:PSS/PEI	ε_b_ = 130%	Current efficiency = 2.0 cd/A	[74]

PU: Polyurethane; PPY: polypyrrole; SWCNT: Single-walled carbon nanotube; P3HT: poly(3-hexylthiophene); PCBM: ((6,6)-phenyl-C_61_-butyric acid methyl ester); PMMA: poly(methylmethacrylate); P3BT: poly(3-butylthiophene); PEN: polyethylene naphthalate.

**Table 2:** Representative achievements made in the field of stretchable sensors.

Application	Material	Mechanical properties	Sensing properties	Reference
Stretchable strain sensors	Graphene woven fabric/PDMS	ε_b_ = 10%	Gauge factor = ~10^6^	[83]
	CNT networks/PU multifilament	ε_b_ = 400%	Gauge factor = ~5	[84]
	AgNWs/PDMS	ε = 0%–50%	Gauge factor = ~1	[60]
	PDMS/AgNWs/PDMS	ε = 0%–70%	Gauge factor = ~5	[89]
Stretchable pressure sensors	PPY-coated PU foam	ε > 1,000%	Sensitivity = 0.0007 mS/N	[93]
	AgNW-embedded PDMS/PMMA	Not stretchable	Sensitivity > 3.8 kPa^−1^	[94]
	Au-coated PDMS micropillars/PANI nanofibers	ε_b_ (biaxial) = 15%	Sensitivity = 2.0 kPa^−1^ Detection limit = 15 Pa	[95]
	Micro-pyramid PDMS/PEDOT:PSS-PUD blend	ε > 40%	Sensitivity = 10.32 kPa^−1^ Detection limit = 23 Pa	[96]
Stretchable temperature sensors	Graphene embedded in PDMS	ε_b_ = 50%	Nonlinear *R versus* *T* in 30–100 °C	[98]
	SWCNT TFT-PANI nanofiber/PET/Ecoflex	ε_b_ (biaxial) = 30%	Sensitivity = 1.0% °C^−1^	[99]
	PDMS/PEDOT:PSS-PUD composite/PU/(R-GO)-PU composite	ε_b_ = 70%	Sensitivity = 1.34% °C^−1^	[100]

TFT: Thin Film Transistor; PANI: polyaniline; PET: poly(ethylene terephthalate).
